# The severity of post-infarction edema suggests a bimodal pattern, whereas the extent does not

**DOI:** 10.1016/j.jocmr.2026.102741

**Published:** 2026-04-28

**Authors:** Robert Jablonowski, David Nordlund, Christos Xanthis, Sebastian Bidhult, Sascha Kopic, Jonathan Berg, Henrik Engblom, Anthony H. Aletras, Håkan Arheden

**Affiliations:** a Department of Clinical Physiology, Clinical Sciences, Lund University and Lund University Hospital, Lund, Sweden; b Laboratory of Computing, Medical Informatics and Biomedical-Imaging Technologies, School of Medicine, Faculty of Health Sciences, Aristotle University of Thessaloniki, Thessaloniki, Greece

**Keywords:** Myocardium at risk, myocardial edema, CMR

## Abstract

**Background:**

Preclinical and clinical data following acute myocardial infarction (MI) have shown conflicting results whether a bimodal pattern of edematous myocardium at risk (MaR) on cardiovascular magnetic resonance (CMR) imaging during the first week exists or not. The objective was to assess the dynamics of MaR following acute experimental MI using a comprehensive CMR protocol.

**Methods:**

Acute MI was induced in seven pigs with reperfusion after 40 min. CMR was performed at baseline, 120 min, 24 h and 7 days post reperfusion. The CMR protocol comprised two T2-mapping sequences, a T2-SSFP sequence (T2-mapping_SSFP_) and a T2-TSE sequence (T2-mapping_TSE_), as well as a T2w short-tau inversion recovery (T2-STIR) sequence. After contrast administration, CE-SSFP and LGE images were acquired. The severity of MaR was defined as the T2 relaxation on T2 maps and the extent of MaR as percentage of edematous myocardium on CE-SSFP and T2-STIR images.

**Results:**

The severity of edema within the MaR was significantly lower at 24 h compared to 120 min (T2 mapping_SSFP_:69±9 vs 80±8 ms; T2 mapping_TSE_:57±7 vs 69±9 ms), followed by a recovery at 7 days (T2 mapping_SSFP_:77±10 ms; T2 mapping_TSE_:67±12 ms). The extent of MaR showed a gradual decrease between 120 min and 24 h on CE-SSFP (29±10 vs 26±9%LVM) and at 7 days compared to both previous time points (CE-SSFP: 24±11%LVM).

**Conclusion:**

The severity of edema within the MaR following acute MI suggests a bimodal pattern, whereas the extent of MaR does not.

## Graphical abstract

**Figure:**
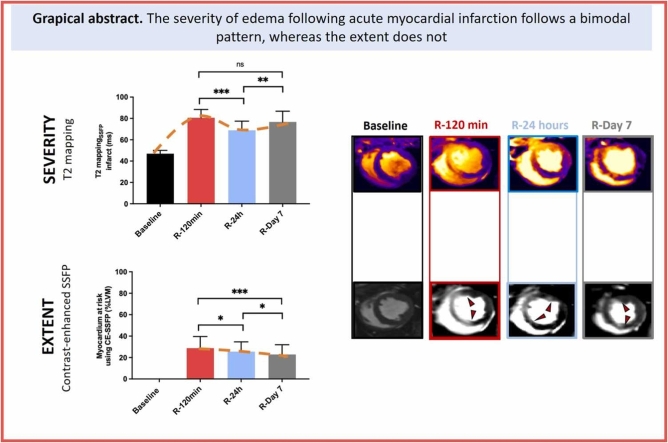

## 1 Introduction

Noninvasive tissue characterization by cardiovascular magnetic resonance (CMR) after myocardial infarction (MI) offers the possibility to evaluate the impact of interventions designed to preserve cardiac function and predict long-term remodeling [1]. Quantification of both infarct size by late gadolinium enhancement (LGE) and extent of edema, as a marker of myocardium at risk (MaR), provides the possibility to calculate myocardial salvage, a surrogate marker for the effect of cardioprotective therapies [2], [3], [4], [5], [6], [7]. In recent years, preclinical and clinical data following acute MI and reperfusion have shown that the severity of edematous MaR on CMR imaging demonstrates a bimodal pattern during the first week [8], [9], [10], [11]. These studies have used T2-mapping as an imaging biomarker of edema. On the other hand, there have also been retrospective data demonstrating that the extent of MaR is stable during the first week in patients using contrast-enhanced steady state free precession (CE-SSFP) [12].

These disparities have been highlighted in a publication from an expert panel on post-MI pathophysiology where it is stated that the use of edema-sensitive CMR sequences as a surrogate for MaR is not recommended given the reported instability of edema post-MI, and also that it possibly can be affected by duration of ischemia and cardioprotective interventions [1]. To date, no study has used both T2-mapping and CE-SSFP imaging to assess the severity and extent of edema post-MI.

Therefore, the aim of this study was to assess the dynamics of edematous MaR during the first week following acute experimental MI. The severity and extent of edema post-MI was studied using a comprehensive CMR protocol, including phantom experiments and finally histopathological validation in a separate cohort.

## 2 METHODS

### 2.1 Experimental protocol

**Fig. 1:**
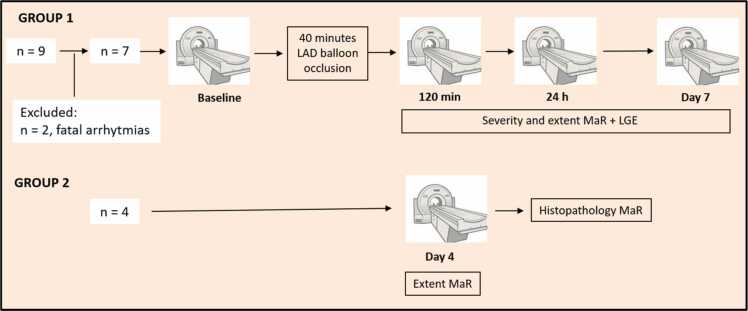
Schematic overview of the study design and cardiovascular magnetic resonance sequences acquired. In group 1, a total of nine pigs were included with two pigs excluded due to fatal arrhythmias. Seven pigs were included in the final analysis and were imaged at baseline followed by 40 min infarct/reperfusion. Serial imaging following reperfusion was performed at 120 min, 24 h and 7 days. In a separate group (group 2) four pigs were imaged at day 4 followed by histopathological assessment of MaR using Evans blue dye. *MaR* myocardium at risk

This study was performed in agreement with the “Guide for the Care and Use of Laboratory Animals” and with approval from the Swedish Agricultural Board (registration number 5.8.18–11702/2019). Part of the experimental design has previously been published [13]. The work flow is shown in Fig. 1. Two groups of pigs were used, group 1 for longitudinal analysis with serial CMR scans and group 2 for histopathological analysis. The animals used were Landrace pigs. All animals were sedated using ketamine (Ketaminol, Intervet, Danderyd, Sweden), midazolam (Dormicum, Roche AB, Stockholm, Sweden), and atropine (Atropin, Mylan, Stockholm, Sweden) by intramuscular injection. Peripheral venous access was established and tracheal intubation was performed. The animals were intubated and connected to a servo-ventilator and anesthesia was maintained with isoflurane delivered by an anesthetic conserving device (AnaConDa, Sedana Medical, Stockholm, Sweden) with addition of fentanyl as required during instrumentation and ischemia/reperfusion. Slow infusions of 5% glucose and Ringers-acetate were started as maintenance fluid after intubation. Introducers were inserted percutaneously via the femoral artery and vein and heparin was administered to prevent clotting. A baseline cardiovascular magnetic resonance imaging (CMR) was then performed. The animals were mechanically ventilated in a supine position during surgical preparation and imaging. After baseline imaging, the animals were transported to a fluoroscopy suite and connected to an intravascular temperature management (IVTM) system (Thermogard XP, ZOLL Circulation, San Jose, California) to maintain the baseline body temperature at 38 C°. A balloon was placed in the LAD distal to the first diagonal and inflated for 40 min to cause myocardial ischemia. During the duration of occlusion, a norepinephrine infusion was titrated as needed to maintain a mean arterial pressure of >60 mmHg and biphasic shocks of 360 J in combination with chest compressions were administered in the event of ventricular fibrillation/tachycardia. The animals were moved back into the CMR scanner after the ischemia/reperfusion period. After 120 min post-reperfusion, a second CMR session was carried out, including sequences to assess ventricular function, edema, and infarct. After the second imaging session, the animals were transported back to the animal facility and awoken. Two more imaging sessions at 24 h and seven days post-reperfusion were carried out with identical anesthetic and CMR protocols as for the 120 min post reperfusion scan. During the experiment, the pigs were monitored using electrocardiogram, plethysmography, heart rate, continuous invasive blood pressure, temperature, EtCO_2_, and blood gas analyses. Following day seven imaging, the animals were terminated by an intravenous injection of Pentobarbital (250 mg/kg, Euthasol vet, Virbac, Kolding, Denmark). Subsequently, the hearts were excised and cut into consecutive 8 mm-thick short-axis slices. Photographs using a digital camera (Nikon, Tokyo, Japan) were taken of all slices and presence of microvascular obstruction was assessed.

### 2.2 Histopathological MaR validation

In a small, separate, cohort of four animals, we performed histopathological validation of MaR using Evans Blue dye, see Fig. 1 (Group 2). These animals were sedated and instrumented similar to the animals described above; however, CMR imaging was only performed at day 4 post-MI. This time point was chosen as LGE and microvascular obstruction has been shown to be stable 5 ± 2 days post MI [1] previous studies have used this time point [8]. Following CMR, animals were re-catheterized with guide catheter placed in the right and left coronary ostium. An over-the-wire balloon was then placed in the LAD distal to the first diagonal and inflated at the same site as the original balloon inflation. Immediately following balloon inflation 20 mL 10% Evans blue dye was injected into both guide catheters to demark the MaR. Subsequently, the animals were euthanized, and the hearts were excised and cut into consecutive 8 mm-thick short-axis slices. Photographs using a digital camera (Nikon, Tokyo, Japan) were taken of all slices.

### 2.3 CMR imaging

#### 2.3.1 Animals

All animals were positioned supine in a 1.5T MR-scanner (MAGNETOM Aera, Siemens Healthineers, Erlangen, Germany) using an 18-channel phased-array chest coil and a spine array coil. During scanning, the animals were mechanically ventilated, and images were acquired at end-expiratory suspended respiration. For baseline imaging, short-axis and long-axis views of the LV were acquired with a steady-state free precession (SSFP) cine sequence as well as T2-mapping. At 120 min, 24 h and 7 days post-reperfusion, the imaging protocol included T2w imaging, T2-mapping and after contrast administration CE-SSFP and late gadolinium enhancement (LGE), see Fig. 1.

#### 2.3.2 T2w imaging and T2 mapping

First, a short-axis stack of T2w short tau inversion recovery (T2 STIR) images were acquired before contrast administration. Following this, a prototype multi-breath-hold T2-SSFP mapping sequence (T2 mapping_SSFP_) which was developed for the study was acquired using 16 different echo times. For comparison, a work in progress (WIP) T2-TSE mapping sequence (T2 mapping_TSE_) was used and designed to mimic the sequence used in prior work [7], [8], [9], [10]. T2-maps were subscribed based on the hyperenhancement seen on the T2-STIR sequence and acquired in a mid-apical ventricular short-axis slice in the same anatomical level at all time points. Standard imaging parameters were: *T2 STIR*: field of view (FOV) 340×276 mm; voxel size 1.3×2.3×8 mm^3^; refocusing flip angle 180°; repetition time (TR) 2RR; echo time (TE) 52 ms. *T2 mapping*_*SSFP*_: FOV 290 × 245; voxel size 1.5×1.7×8 mm^3^; SSFP flip angle 70°; TR 2RR; and 16 echo times ranging from 25 to 200 ms. *T2 mapping*_*TSE*_: FOV 360×270 mm; voxel size 1.4×1.5×8 mm^3^; refocusing flip angle 180°; TR 2RR; and 10 echo times ranging from 6.9 to 69 ms.

#### 2.3.3 Contrast-enhanced SSFP

A contrast-enhanced balanced SSFP (CE-SSFP) cine sequence, covering both short and long-axis views, was acquired to quantify ventricular function and myocardium at risk (MaR) [14], [15]. Cine images were acquired with retrospective ECG gating at end-expiratory breath holds, 6 min after the injection of 0.2 mmol/kg gadolinium (Gd)-DOTA (Dotarem, Guerbet, Roissy, France). Standard parameters were: TR 2.8 ms; TE 1.4 ms; flip angle 60°; FOV 320×290 mm; voxel size 1.7×1.7×8 mm with no slice gap and 25 reconstructed time frames.

#### 2.3.4 Late gadolinium enhancement

Both long- and short-axis LGE images were acquired 15–18 min post Gd-contrast injection, to visualize infarct size in-vivo. A TI-scout was used to estimate the inversion time to null normal myocardium. The LGE sequence used the following parameters: TR 2.8 ms; TE 1.2 ms; flip angle 50°; FOV 360×270 mm; voxel size 1.4×1.4×8.

#### 2.3.5 Phantom data

We compared the T2-SSFP prototype sequence to a WIP T2-TSE and a single-echo Spin-echo reference standard T2 sequence in a gel phantom with T1/T2 resembling edematous myocardium at 1.5 Tesla (T1: 1200 ms; T2: 60 ms). The reference standard single-echo Spin echo T2 sequence had a repetition time longer than 5xT1 (TR = 10 seconds) and used 10 echo times (8, 10, 30, 60, 80, 100, 150, 200, 500, 1000 ms) to estimate T2.

### 2.4 Image analysis

Analysis of CMR images was performed using the software Segment 3.1 (Medviso, Lund, Sweden) [16] and analysis was blinded to time points. For T2 mapping, regions of interest were manually drawn in all individual images with different echo times and a 3-parameter model for T2 relaxation was used to map T2 values [17], [18]. The goodness-of-fit for T2 mapping_SSFP_ was high, with a mean coefficient of determination (R²) of 0.95±0.02 across all time points. In the T2w, CE-SSFP and LGE images left ventricular endo- and epicardial contours were manually delineated in the short-axis images with exclusion of papillary muscles and trabeculae. Left ventricular mass and volumes were derived from cine images. The severity of MaR was defined as the T2 relaxation (ms) from T2 mapping sequences and measured by drawing a region of interest (ROI) in the infarct area within the edematous MaR at all time points post reperfusion. Also, ROIs were drawn in the salvaged MaR outside of the infarct. T2 values in remote myocardium at all time points were also obtained. All ROIs in the infarct area, salvaged MaR outside of the infarct and remote myocardium were drawn by guidance and co-registration of LGE and CE-SSFP images. Care was taken to place ROIs within a single tissue type (infarct, salvaged MaR, remote) and avoid signal contamination from adjacent tissue types and blood. Any hypointense areas suggestive of microvascular obstruction or hemorrhage were excluded in the ROIs for T2 quantification. The extent of MaR was defined as percentage of edematous myocardium and manually delineated in T2 STIR and CE-SSFP images by identifying hyperintense areas, including areas of microvascular obstruction (MVO) or intramyocardial hemorrhage. Infarct and MVO were quantified on LGE images at all time points using a validated semi-automatic method [19], [20]. The placement of ROIs was performed by an experienced reviewer (RJ: 11 years of CMR experience).

The histopathology slices, stained with Evans blue were evaluated by delineating the endocardium, epicardium and MaR in software Segment. Myocardium at risk was expressed as % MaR per slice. Paired CE-SSFP slices were then delineated as described previously.

### 2.5 Statistics

Statistical analyses were performed using the software GraphPad Prism v. 8.2.1. (GraphPad Software, Inc., San Diego, California) and Stata software (version 18.0, StataCorp, College Station, Texas). Continuous data are presented as mean ± SD. Longitudinal changes across time points were analyzed using linear mixed-effects models with animals as a random intercept and time as a categorical fixed effect to account for within-animal repeated measures. Model estimates are reported as mean differences with 95% confidence intervals and corresponding p-values. The primary endpoint was defined as T2mapping_SSFP_ at 24 h, while other variables were treated as secondary outcomes. Multiplicity for secondary endpoints was addressed using Holm correction. Pairwise comparisons between different T2 mapping sequences at each time point were derived from estimated marginal means. A two-sided p-value <0.05 was considered statistically significant. In a subset of animals (n=4), interobserver agreement (CE-SSFP, T2-STIR, and T2 mapping_SSFP_) and intra-observer agreement (T2 mapping_SSFP_) were assessed for all time points using both Bland–Altman analysis (mean ± 1.96 SD), and the intraclass correlation coefficient (ICC), calculated using a two-way random-effects model with absolute agreement for single measurements (ICC(2,1)). Sample size was chosen in accordance to previously published data in the field [8], [9]. The final cohort comprised seven animals, which limit power for detecting small differences between time points. We therefore emphasize effect sizes with 95% confidence intervals to reflect statistical precision. Based on the observed within-subject variability, the study is powered primarily to detect moderate-to-large longitudinal changes. The manuscript was written according to the Animal Research: Reporting of In-Vivo Experiments (ARRIVE) guidelines [21].

## 3 RESULTS

### 3.1 Baseline characteristics

Out of nine animals, two did not complete the study protocol due to fatal arrhythmias prior to reperfusion and were therefore not included in the final analysis. The animals’ temperature at reperfusion was 37.9±0.3°C. Descriptive baseline and CMR data are presented in Table 1 and Supplemental Table 1. Details on hemodynamic and experimental parameters are presented in Supplemental materials and Supplemental Tables 2 and 3.

**Table 1:** Baseline and CMR characteristics

	***Baseline***	***R-120min***	***R-24h***	***R-Day 7***
*Sex [n, %female]*	3, 43%			
*Weight [kg]*	39±3			
*EDV [mL]*	110±8	110±13	110±12	110±10
*ESV [mL]*	55±6	61±15	63±14	56±10
*SV [mL]*	58±5	47±10	44±9	56±8
*EF [%]*	51±3	44±9	41±9	50±7
*Cardiac Output [l/min]*	4.7±0.5	3.7±1.1	3.2±0.4	5.2±0.8
*Infarct size [%LVM]*		19±9	18±8	15±8
*Myocardium at risk CE-SSFP [%LVM]*		29±11	26±9	23±9
*Myocardium at risk T2-STIR [%LVM]*		29±10	26±10	22±8
*Microvascular obstruction [%LVM]*		2±2	2±1	2±2
*Myocardial salvage [%]*		38±13	36±16	37±14

*EDV* end-diastolic volume, *ESV* end-systolic volume, *SV* stroke volume, *EF* ejection fraction, *LVM* left ventricular mass, *CE-SSFP,* contrast-enhanced steady state free precession

### 3.2 Phantom data

In phantom experiments, both the T2-SSFP prototype and T2-TSE resulted in T2 estimates similar to Spin echo: T2 Spin echo (reference): 60.0 ± 0.5 ms; T2-SSFP: 60.6 ± 3.0 ms; T2-TSE: 60.9 ± 0.7 ms.

### 3.3 Histopathology

**Fig. 2:**
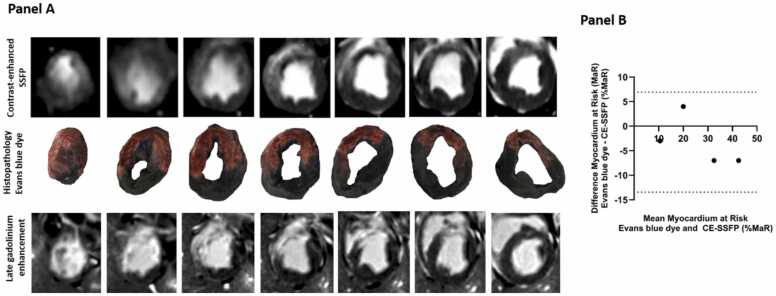
In panel A, an example depicting MaR using CMR contrast-enhanced SSFP images (top row) and histopathological staining with Evans blue dye (middle row) and late gadolinium enhancement images is shown. In the middle row blue areas represent the remote myocardium and reddish area the MaR. In panel B, the relationship between myocardium at risk (MaR) using histopathology with Evans blue dye and CMR contrast-enhanced SSFP images. Bland–Altman analysis demonstrates a bias of 3±5%MaR. Dashed lines = 95% limits of agreement. *MaR* myocardium at risk, *CMR* cardiovascular magnetic resonance

In the separate group imaged only at day 4 post reperfusion, the area of MaR using Evans blue dye was similar to MaR by CE-SSFP (bias 3 ± 5% area MaR, 95% limits of agreement −13,4 to 6,9), see Fig. 2B. For transparency, corresponding LGE images are also shown in Fig. 2A and infarct size by LGE was smaller than MaR by Evans blue as expected (bias −14 ± 8).

In the longitudinal imaged group, five pigs had microvascular obstruction on excised hearts, which was consistent with in vivo LGE imaging at 2 h, 24 h and 7d, see Supplemental Figure 1.

### 3.4 Severity of edema

**Fig. 3:**
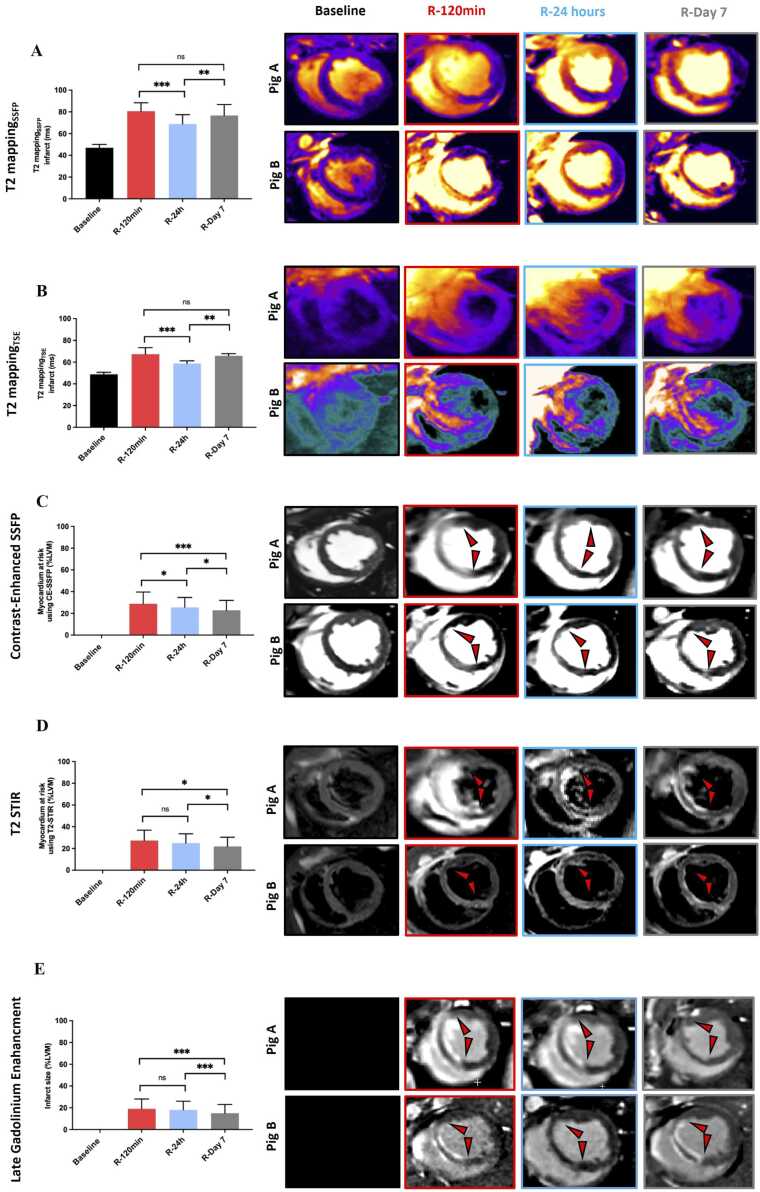
This figure shows imaging at baseline and following ischemia/reperfusion at three time points with measures of severity of edema in myocardium at risk (MaR) using two T2- mapping sequences (panels A and B) and measures of extent of MaR using contrast-enhanced SSFP (panel C), T2 STIR (panel D) and infarct size by late gadolinium enhancement imaging (panel E). Two examples are shown from two different animals. **A**. T2 relaxation times using a T2-SSFP mapping sequence (T2 mapping_SSFP_). There is a significantly lower T2 at 24 h compared to 120 min and seven days. **B.** T2 relaxation times using a T2-TSE mapping sequence (T2 mapping_TSE_). Similar results are seen as for T2 mapping_SSFP_ in panel A. **C.** Extent of MaR using CE-SSFP demonstrates that there is no bimodal pattern of the extent of MaR over seven days but rather a decline in extent of MaR from 2 h to seven days post reperfusion. **D.** Extent of MaR using T2-STIR demonstrates similar results as CE-SSFP in panel C. **D.** Infarct size by late gadolinium enhancement imaging demonstrating smaller infarct day 7 compared to other time points. Statistical significance was determined using Holm-adjusted pairwise comparisons based on linear mixed-effects models * = p<0.05; ** = p<0.01; *** = p<0.001. R = Reperfusion. *CE-SSFP* contrast-enhanced steady state free precession

Fig. 3A shows the severity of edema within the MaR from T2 mapping SSFP. T2 values demonstrated a significant effect of time in the mixed-effects model (p < 0.001). T2 values were significantly higher at all time points compared to baseline. Between post-injury time points, T2 mappingSSFP decreased from 2 h to 24 h (95% CI −19 to −8.3; p<0.001), whereas no significant difference was observed between 2 h and 7 days after adjustment (95% CI −9.2 to −1.3; p = 0.14). T2 values at 7 days remained significantly higher than at 24 h (95% CI −15 to −4.4; p = 0.002). T2 values obtained using the TSE sequence demonstrated significant temporal changes (Fig. 3B). Compared with baseline, T2-TSE was significantly increased at 2 h, 24 h, and 7 days (p<0.001). T2-TSE peaked at 2 h and decreased significantly by 24 h (95% CI −13 to −5.3; p<0.001). Thereafter, T2-TSE increased again between 24 h and 7 days (95% CI 3.0–10.8; p = 0.0060). When comparing the two T2 mapping sequences, T2 mappingSSFP yielded higher T2 values at all time points post reperfusion in both infarcted and salvaged MaR compared to T2 mappingTSE (p<0.05 for all). In remote myocardium there was no difference between time points using either T2 mappingSSFP or T2 mappingTSE (p>0.05 for all). For T2 mappingSSFP, interobserver and intra-observer variability was 0.3 **±** 5.1 ms (ICC 0.96*; CI: 0.86–0.99*) and 0.1 **±** 2.4 ms (ICC 0.98*; 0.95–1.00*), respectively. See Table 2 for all T2 mapping relaxation times and all mean differences with 95% confidence intervals, and Holm-adjusted p-values for all pairwise time-point comparisons are provided in Supplementary Table 4.

**Table 2:** T2 relaxation times from remote, salvaged myocardium at risk (MaR) and infarct

	**T2 mapping**_**SSFP**_				**T2 mapping**_**TSE**_			
	*Baseline*	*R-120min*	*R-24h*	*R-Day 7*	*Baseline*	*R-120min*	*R-24h*	*R-Day 7*
*Remote (ms)*	47±3	50±2	51±3	55±4	49±3	50±3	51±5	52±3
*Salvaged MaR (ms)*	-	70±9	64±7	65±7	-	62±7	55±6	58±7
*Infarct*	-	80±8	69±9	77±10	-	69±9	57±7	67±12

### 3.5 Extent of edema

Myocardium at risk (MaR) assessed by CE-SSFP (Fig. 3C) showed a reduction in extent between 120 min and 24 h (95% CI 1.2–5.0; p = 0.016) and between 24 h and 7 days (95% CI −4.8 to 0.96; p = 0.021). For MaR assessed by T2-STIR (Fig. 3D) decreased over time with a significant reduction from 24 h to 7 days (95% CI −8.6 to 1.7; p = 0.024), while the change between 2 h and 24 h was not statistically significant (95% CI −3.6 to −3.4; p = 0.96). All pairwise time-point comparisons are provided in Supplemental Table 1. The interobserver variability for MaR by CE-SSFP was −0.3 ± 2.8%LVM (ICC 0.97; CI: 0.89–0.99) and for T2-STIR −1.0 ± 3.9%LVM (ICC 0.92; CI: 0.73–0.98).

### 3.6 Infarct size, MVO, and myocardial salvage

Infarct size by LGE decreased significantly over the course of one week (Fig. 3E, p < 0.05 for all) but there was no difference in myocardial salvage over one week, see Table 1 and Supplemental Table 1. MVO was seen in five pigs in the longitudinal group, and the size of MVO did not change from 2 h to 7 days on LGE imaging, see Table 1. The pigs with MVO had similar edema extent and severity dynamics as those without, however interpretation is limited by the small sample size of the no MVO group (see Supplemental Figure 2).

## 4 DISCUSSION

This study has demonstrated that the severity of edema after acute experimental ischemia/reperfusion injury is suggestive of a bimodal pattern with a reduction in T2 at 24 h post reperfusion that rises again at 7 days. The extent of edematous MaR did not follow a bimodal pattern but rather a decline from 24 h to 7 days post reperfusion. Our study, utilizing phantom T2 data, and a comprehensive longitudinal CMR protocol, has demonstrated that the pathophysiological changes following acute myocardial infarction may appear differently depending on CMR sequences used.

### 4.1 Relation to prior studies

#### 4.1.1 Severity of edema

Our work supports a bimodal pattern with a drop in severity of edema within the MaR from 120 min to 24 h post reperfusion [8], [9], [10], [11]. However, we still see a significant difference between the severity in T2 between 24 h and baseline, suggesting that the edema is still present even though it is less severe. In previous studies using T2 mapping, it has been shown that edema at 24 h after reperfusion declines significantly and in porcine models even below measured baseline T2 values [8]. In the present study, we used two different T2 mapping sequences with the idea to 1) mimic previously used sequences [8], [9], [10], [11] using T2 mapping_TSE_ and 2) to introduce a more robust sequence, T2 mapping_SSFP_, with more echo times rendering a more densely sampled T2-decay. The use of T2 mapping techniques has the advantage of being directly quantitative and providing measures of the severity of edema. However, caution should be taken when interpreting the results since different T2 mapping pulse sequences may yield different absolute values. Due to the exclusion of hypointense potential hemorrhagic regions in our study, the measured T2 severity might be higher than if these regions were included [11].

#### 4.1.2 Extent of edema

In previous studies on longitudinal assessment of edema after MI, T2w CMR imaging has mainly been used [8], [9], [10], [11], [22], [23]. T1 mapping has also been used [24] demonstrating that intensity of edema is bimodal (imaging at 3 h, 24 h, 6 days) with an unchanged extent. Furthermore, CE-SSFP has been used in retrospective analysis of the extent of edema in patients but serial imaging in the same patient was not performed during the first week [12]. Recently, the mechanism behind contrast enhancement in CE-SFFP images experimentally and clinically was investigated [25]. To our knowledge this is the first study to use both CE-SSFP and T2w IR imaging in this context of serial imaging after acute MI during the first week. Previously it has been shown that the extent of edematous MaR by T2 mapping_TSE_ disappears at 24 h post reperfusion only to appear again at 4 days post reperfusion [8]. This phenomenon has been shown both experimentally and in patients and may be partly affected by bleeding within the infarcted area with elevated amounts of hemoglobin and myoglobin affecting T2 values within the injured myocardium [22], [26]. In our study, five pigs in the longitudinal group had MVO evident on excised, sliced, hearts and this was consistent with findings on in vivo LGE imaging. The severity and extent of edema was similar in pigs with and without MVO. However, the areas of MVO were small, and larger MVO could possibly affect edema dynamics.

Furthermore, windowing of images for analysis of extent of MaR is necessary and should be done with caution since it can impact results for both T2w and CE-SSFP imaging. Previous external validation data by Hansen et al. comparing CE-SSFP-derived MaR with Evans blue histopathology in a separate cohort at day 7–10 after reperfusion demonstrated a similarly low bias [27]. Although broadly consistent with our findings on day four, extrapolation across these time points should be interpreted with caution given potential temporal changes.

### 4.2 Physiological relevance

There have been numerous studies, both experimental and clinical, quantifying edema following acute MI at only one time point [3], [4], [5], [28], [29], [30], [31], [32], [33]. Longitudinal studies of both the extent (anatomical volume) and severity of edema yield interesting physiological aspects to consider. The bimodal description of edema has been attributed to a first wave of edema due to reperfusion injury and the second wave due to influx of inflammatory cells and tissue healing [9]. In the current study, assessment of water content in the myocardium using a desiccation method was not performed to serve as a gold standard for the severity of edema. However, it has previously been shown using desiccation that water content also followed a bimodal pattern, however at 24 h, edema did not return to baseline as has been shown for T2 values [8]. In the present study, none of the T2 mapping sequences returned to baseline at 24 h, instead they followed the same pattern as the “gold standard” of residual water content using the desiccation method. Furthermore, edema was visually evident with both contrast (CE-SSFP) and non-contrast sequences (T2 STIR) at this time point. It has previously been hypothesized that the water content of a given volume of myocardium can change (i.e., severity) without a change in the overall volume of the affected myocardium (i.e., extent), which may serve as a mechanistic explanation to our findings [24].

### 4.3 Clinical relevance

Our results imply that a standardization of CMR protocol is of importance when imaging edema post MI. It is also important to use multiple sequences to depict both the severity and extent of edema, including CE-SSFP and T2-mapping. Furthermore, even though we did not perform imaging between 24 h and 7 days, we support the proposition that in cardioprotective trials, the edematous MaR and infarct should not be imaged early (≤ 24 h) after infarction as edematous changes are dynamic [1]. A key finding in our study is that the extent of edema does not show a bimodal pattern and instead demonstrates a decrease over 7 days. However, there is only a 3-percentage point decrease in extent from 24 h to 7 days, which are the two time points where clinical imaging could be feasible. Also, myocardial salvage did not change over the studied timepoints during the first week after infarction*.* Currently, LGE-based infarct size measurements, however, remain the surrogate outcome measure of choice when designing cardioprotective studies [1]. The T2-mapping_SSFP_ sequence used in the current study is not clinically applicable due to long breath-holds and acquisition time. The translational relevance of our study should be viewed in the light of previous translational research where dynamics of edema have been stable from large animal I/R models to clinical studies [8], [10]. The porcine I/R model used with 40 min of occlusion has been carefully chosen to mimic a clinical scenario where approximately 50% of the area at risk becomes necrotic [34].

## 5 Limitations

The custom 16-echo T2-SSFP prototype used in the current study is not clinically available which limits the generalizability. Also, no CMR imaging was performed between 24 h and day 7, due to logistical challenges and we opted to perform imaging on day 4 in a separate cohort of pigs. This time period, however, has been less controversial than 24 h post revascularization. As this study used an infarct/reperfusion model, our results cannot be extrapolated to non-reperfused MI. T2 mapping was performed in a single mid-apical slice, whereas extent was quantified from full short-axis stacks which may yield sampling mismatch as the exact same slice is not compared for extent and severity analysis. Potential sources of variability between imaging sessions include physiological parameters, however there was no difference in blood pressure, heart rate, temperature, or weight. However, the single left ventricular slice imaged for T2 severity analysis may differ slightly between imaging sessions which is an inherent limitation of a longitudinal study design. We did not specifically assess intramyocardial hemorrhage using T2* or histologically, which could be a part of the explanation of the bimodal peak in edema severity. We did also not use a direct method do assess myocardial water content, such as desiccation, however we included a subset of animals where we assessed the extent of MaR histopathologically using Evans blue dye. Interobserver and intra-observer variability data was only performed in a subset of animals (n=4).

## 6 CONCLUSION

The severity of edema following acute myocardial infarction suggests a bimodal pattern with a drop at 24 h post reperfusion, whereas the extent of edema does not. Absolute T2 values differ between T2 mapping sequences and standardization of a CMR protocol for the assessment of MaR by T2 mapping is therefore of importance.

## Disclosures

H.A. is stockholder in Imacor AB, Lund, Sweden. H.E. has received consultancy fees from Imacor AB for analysis of cardiac MRI. C.X and A.H.A are stockholders in Corsmed AB.

## Author contributions

**Robert Jablonowski:** Writing – review & editing, Writing – original draft, Project administration, Formal analysis, Data curation, Conceptualization. **David Nordlund:** Writing – review & editing, Project administration, Data curation. **Christos Xanthis:** Writing – review & editing, Project administration, Data curation. **Sebastian Bidhult:** Writing – review & editing, Project administration, Data curation. **Sascha Kopic:** Writing – review & editing, Project administration, Data curation. **Jonathan Berg:** Writing – review & editing, Project administration, Data curation. **Henrik Engblom:** Writing – review & editing, Supervision, Project administration, Conceptualization. **Anthony H. Aletras:** Writing – review & editing, Supervision, Data curation, Conceptualization. **Håkan Arheden:** Writing – review & editing, Supervision, Project administration, Funding acquisition, Conceptualization.

## Availability of data and materials

The data underlying this article will be shared on reasonable request to the corresponding author

## Declaration of competing interests

The authors declare the following financial interests/personal relationships which may be considered as potential competing interests: Hakan Arheden reports financial support was provided by Swedish Heart and Lung Association. Hakan Arheden reports financial support was provided by Skåne University Hospital Lund. Hakan Arheden reports a relationship with Imacor AB that includes: equity or stocks. Henrik Engblom reports a relationship with Imacor AB that includes: consulting or advisory. Christos Xanthis reports a relationship with Corsmed AB that includes: equity or stocks. Anthony H Aletras reports a relationship with Corsmed AB that includes: equity or stocks. The remaining authors declare that they have no known competing financial interests or personal relationships that could have appeared to influence the work reported in this paper.
