# Supplementary material for: The severity of post-infarction edema suggests a bimodal pattern, whereas the extent does not
Source: J Cardiovasc Magn Reson. 2026 Apr 28;28(1):102741. doi: 10.1016/j.jocmr.2026.102741 (PMC13241639; doi:10.1016/j.jocmr.2026.102741)
Supplement: Supplementary file 1 — Supplementary material [file mmc1.docx]

**Supplemental material**

**Microvascular obstruction**

**
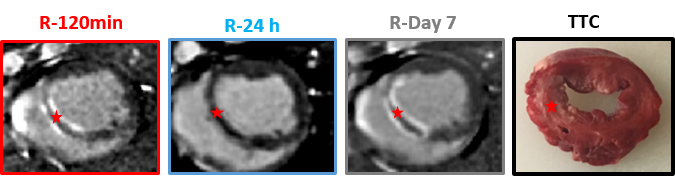
*Supplemental Figure 1.*** *Example of* *microvascular obstruction (red asterix) on in vivo LGE images 120minutes, 24 hours and 7 days post reperfusion and on corresponding triphenyl tetrazolium chloride (TTC) stained slice.*

The edema severity dynamics over time using mixed-effects modeling did not indicate a differential temporal pattern between groups with or without microvascular obstruction (MVO), with both demonstrating a reduction at 24 hours and recovery at 7 days. The same pattern for both groups was also seen for edema extent using CE-SSFP. Interpretation is limited by the small sample size of the no MVO group (n=2).

***
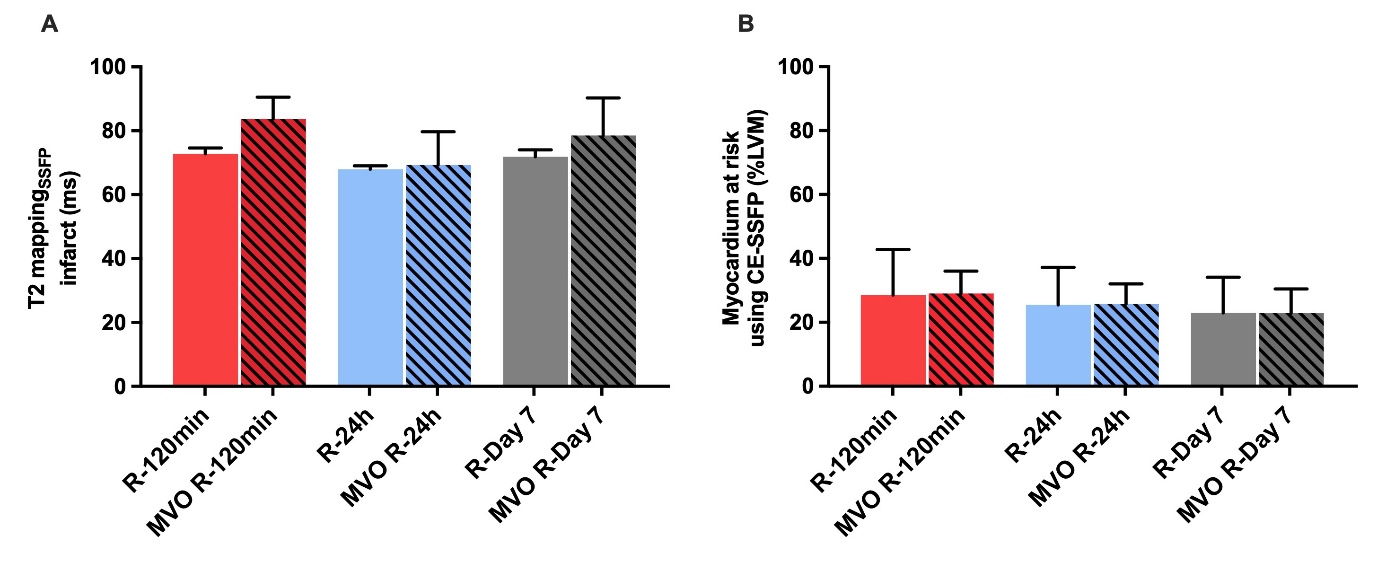
***

***Supplemental Figure 2.*** *The figure shows the* *severity of edema (****panel A****) and extent of edema (****panel B****) in pigs with (striped columns) and without microvascular obstruction at 120 minutes, 24 hours and 7 days post reperfusion. The edema severity and extent dynamics over time did not differ between groups with or without MVO.*

**Supplemental Table 1.** Pairwise time-point comparisons using mixed model analysis.

| Parameter | Comparison | Mean diff | 95% CI | p | pHolm |
| --- | --- | --- | --- | --- | --- |
| EDV (ml) | 24h vs 7d | -5.69 | [-12.11, 0.73] | 0.08 | 0.40 |
| EDV (ml) | 2h vs 24h | 2.12 | [-4.30, 8.53] | 0.50 | 1.00 |
| EDV (ml) | 2h vs 7d | -3.58 | [-10.00, 2.83] | 0.25 | 1.00 |
| EDV (ml) | Baseline vs 24h | 12.02 | [5.61, 18.44] | <0.001 | 0.002 |
| EDV (ml) | Baseline vs 2h | 14.14 | [7.72, 20.55] | <0.001 | <0.001 |
| EDV (ml) | Baseline vs 7d | 8.44 | [2.03, 14.85] | 0.01 | 0.04 |
| ESV (ml) | 24h vs 7d | 15.68 | [4.64, 26.73] | 0.01 | 0.03 |
| ESV (ml) | 2h vs 24h | 6.55 | [-4.50, 17.60] | 0.23 | 0.69 |
| ESV (ml) | 2h vs 7d | 22.23 | [11.18, 33.28] | <0.001 | 0.001 |
| ESV (ml) | Baseline vs 24h | 13.00 | [1.95, 24.05] | 0.03 | 0.08 |
| ESV (ml) | Baseline vs 2h | 19.55 | [8.50, 30.60] | 0.002 | 0.006 |
| ESV (ml) | Baseline vs 7d | -2.68 | [-13.73, 8.37] | 0.61 | 0.61 |
| SV (ml) | 24h vs 7d | -18.08 | [-29.13, -7.03] | 0.004 | 0.01 |
| SV (ml) | 2h vs 24h | 4.56 | [-6.49, 15.61] | 0.39 | 1.00 |
| SV (ml) | 2h vs 7d | -13.52 | [-24.57, -2.47] | 0.02 | 0.06 |
| SV (ml) | Baseline vs 24h | 8.48 | [-2.57, 19.53] | 0.12 | 0.35 |
| SV (ml) | Baseline vs 2h | 13.04 | [1.99, 24.09] | 0.03 | 0.08 |
| SV (ml) | Baseline vs 7d | -0.48 | [-11.53, 10.57] | 0.93 | 0.93 |
| EF (%) | 24h vs 7d | -17.33 | [-28.57, -6.09] | 0.006 | 0.02 |
| EF (%) | 2h vs 24h | -5.04 | [-16.28, 6.20] | 0.33 | 1.00 |
| EF (%) | 2h vs 7d | -22.37 | [-33.61, -11.13] | <0.001 | <0.001 |
| EF (%) | Baseline vs 24h | -6.84 | [-18.08, 4.40] | 0.22 | 0.65 |
| EF (%) | Baseline vs 2h | -11.88 | [-23.12, -0.64] | 0.04 | 0.12 |
| EF (%) | Baseline vs 7d | 10.49 | [-0.75, 21.73] | 0.06 | 0.19 |
| Cardiac Output (L/min) | 24h vs 7d | -2.05 | [-2.77, -1.33] | <0.001 | <0.001 |
| Cardiac Output (L/min) | 2h vs 24h | 0.27 | [-0.45, 0.99] | 0.42 | 1.00 |
| Cardiac Output (L/min) | 2h vs 7d | -1.78 | [-2.50, -1.06] | <0.001 | <0.001 |
| Cardiac Output (L/min) | Baseline vs 24h | 1.35 | [0.63, 2.07] | <0.001 | 0.002 |
| Cardiac Output (L/min) | Baseline vs 2h | 1.62 | [0.90, 2.34] | <0.001 | <0.001 |
| Cardiac Output (L/min) | Baseline vs 7d | -0.70 | [-1.42, 0.02] | 0.06 | 0.11 |
| Infarct size (%LVM) | 24h vs 7d | 2.92 | [1.53, 4.31] | <0.001 | <0.001 |
| Infarct size (%LVM) | 2h vs 24h | 1.61 | [0.22, 3.00] | 0.03 | 0.06 |
| Infarct size (%LVM) | 2h vs 7d | 4.53 | [3.14, 5.92] | <0.001 | <0.001 |
| Myocardium at risk CE-SSFP (%LVM) | 24h vs 7d | 2.87 | [0.96, 4.78] | 0.007 | 0.02 |
| Myocardium at risk CE-SSFP (%LVM) | 2h vs 24h | 3.09 | [1.18, 5.00] | 0.005 | 0.02 |
| Myocardium at risk CE-SSFP (%LVM) | 2h vs 7d | 5.96 | [4.05, 7.87] | <0.001 | <0.001 |
| Myocardium at risk T2-STIR (%LVM) | 24h vs 7d | 5.14 | [1.65, 8.63] | 0.008 | 0.02 |
| Myocardium at risk T2-STIR (%LVM) | 2h vs 24h | -0.09 | [-3.58, 3.40] | 0.96 | 0.96 |
| Myocardium at risk T2-STIR (%LVM) | 2h vs 7d | 5.06 | [1.57, 8.55] | 0.009 | 0.03 |
| Microvascular obstruction (%LVM) | 24h vs 7d | -0.21 | [-1.48, 1.05] | 0.70 | 0.70 |
| Microvascular obstruction (%LVM) | 2h vs 24h | 0.72 | [-0.55, 1.99] | 0.25 | 0.50 |
| Microvascular obstruction (%LVM) | 2h vs 7d | 0.50 | [-0.77, 1.77] | 0.41 | 0.70 |
| Myocardial salvage (%) | 24h vs 7d | -1.94 | [-10.86, 6.98] | 0.64 | 0.64 |
| Myocardial salvage (%) | 2h vs 24h | -0.41 | [-9.33, 8.51] | 0.92 | 0.92 |
| Myocardial salvage (%) | 2h vs 7d | -2.36 | [-11.28, 6.56] | 0.58 | 0.64 |

***Differences between pigs with and without defibrillation shocks***

*Defibrillation shocks were necessary in 5 out 7 animals after occlusion and during early reperfusion. A linear mixed-effects model with animal as a random intercept and time and group was used to assess differences. For infarct size there was no main effect of group (p = 0.286) and no significant group × time interaction (p > 0.3), indicating a similar temporal pattern in both groups.*

*The severity of edema from T2 mapping_SSFP_ showed a similar bimodal pattern over time with significant time-dependent changes in both groups. In the group with shocks values were higher at 2h, 24h and 7d compared with baseline (all p<0.001), with 24h lower than 2h (p=0.001) and 7d higher than 24h (p=0.004). In Group 2, all time-point comparisons were statistically significant, however, inference is limited due to the small sample size.*

*The extent of myocardium at risk (MaR) using CE-SSFP demonstrated a significant decrease over time and there were no significant main effect of group (p=0.54) and no significant group×time interaction (p>0.60), indicating a similar temporal pattern in both groups.*

*Microvascular obstruction (MVO) was present in three pigs with shocks and in two without.*

***Supplemental Table 2.***

|  | *With defibrillation shocks* | | | | *Without defibrillation shocks* | | | |
| --- | --- | --- | --- | --- | --- | --- | --- | --- |
|  | *Baseline* | *R-120min* | *R-24h* | *R-Day 7* | *Baseline* | *R-120min* | *R-24h* | *R-Day 7* |
| *Infarct size (% LVM)* | *-* | *20 ± 10* | *19 ± 9* | *17 ± 9* | *-* | *12 ± 2* | *11 ± 3* | *8 ± 2* |
| *T2 mapping_SSFP_ edema severity (ms)* | *47 ± 3* | *81 ± 7* | *70 ± 4* | *79 ± 10* | *48 ± 5* | *80 ± 7* | *61 ± 7* | *70 ± 9* |
| *Extent MaR (% LVM)* | *-* | *30 ± 12* | *27 ± 9* | *25 ± 10* | *-* | *25 ± 1* | *22 ± 1* | *18 ± 2* |
| *Microvascular obstruction (n)* | *-* | *3* | | | *-* | *2* | | |

**Norepinephrine**

Norepinephrine was required in all animals to maintain a mean arterial pressure above 60mmHg during occlusion and early reperfusion, the infusion rate started at 0.05ug/kg/min and was titrated as needed.

**Supplementary Table 3.** Physiological parameters

| Parameter | Baseline | 2h | 24h | 7d |
| --- | --- | --- | --- | --- |
| Systolic blood pressure (mmHg) | 90 ± 15 | 88 ± 13 | 82 ± 12 | 86 ± 5 |
| Diastolic blood pressure (mmHg) | 48 ± 7 | 47± 8 | 45 ± 8 | 44 ± 3 |
| Mean arterial pressure (mmHg) | 63 ± 10 | 61 ± 9 | 57 ± 9 | 58 ± 3 |
| Weight (kg) | 39 ± 3 | 39 ± 3 | 39 ± 3 | 39 ± 3 |
| Temperature (°C) | 38 ± 1 | 38 ± 0.3 | 38 ± 0.2 | 38 ± 0.3 |
| Heart rate (bpm) | \| 82 ± 9 \| \| --- \| | \| 78 ± 22 \| \| --- \| | \| 75 ± 9 \| \| --- \| | \| 94 ± 10 \| \| --- \| |

All pairwise time comparisons above using mixed-effects model with Holm-adjusted p-values were non-significant.

**Supplemental Table 4.** *T2* mapping *relaxation times*

| Parameter | Comparison | Mean diff | 95% CI | p | p_adjusted |
| --- | --- | --- | --- | --- | --- |
| T2 mappingSSFP (ms) | 24h vs 7d | -9.7 | [-14.9, -4.4] | <0.001 | 0.002 |
| T2 mappingSSFP (ms) | 2h vs 24h | 13.7 | [8.3, 18.8] | <0.001 | <0.001 |
| T2 mappingSSFP (ms) | 2h vs 7d | 3.9 | [-1.3, 9.2] | 0.137 | 0.14 |
| T2 mappingSSFP (ms) | Baseline vs 24h | -20.0 | [-24.8, -14.3] | <0.001 | <0.001 |
| T2 mappingSSFP (ms) | Baseline vs 2h | -33.1 | [-38.4, -27.9] | <0.001 | <0.001 |
| T2 mappingSSFP (ms) | Baseline vs 7d | -29.2 | [-34.5, -23.9] | <0.001 | <0.001 |
|  |  |  |  |  |  |
| T2 mappingTSE (ms) | 24h vs 7d | -6.9 | [-10.8, -3.0] | 0.002 | 0.006 |
| T2 mappingTSE (ms) | 2h vs 24h | 9.2 | [5.3, 13.1] | <0.001 | <0.001 |
| T2 mappingTSE (ms) | 2h vs 7d | 2.3 | [-1.6, 6.2] | 0.227 | 0.23 |
| T2 mappingTSE (ms) | Baseline vs 24h | -8.9 | [-12.8, -5.1] | <0.001 | <0.001 |
| T2 mappingTSE (ms) | Baseline vs 2h | -18.2 | [-22.0, -14.2] | <0.001 | <0.001 |
| T2 mappingTSE (ms) | Baseline vs 7d | -15.9 | [-19.7, -11.9] | <0.001 | <0.001 |
